# The Interaction of EphA4 With PDGFRβ Regulates Proliferation and Neuronal Differentiation of Neural Progenitor Cells *in vitro* and Promotes Neurogenesis *in vivo*

**DOI:** 10.3389/fnagi.2020.00007

**Published:** 2020-02-11

**Authors:** Qingfa Chen, Hao Song, Chuanguo Liu, Jun Xu, Chuanfei Wei, Wei Wang, Fabin Han

**Affiliations:** ^1^The Institute for Tissue Engineering and Regenerative Medicine, The Liaocheng University/Liaocheng People’s Hospital, Liaocheng, China; ^2^The Translational Research Laboratory of Stem Cells and Traditional Chinese Medicine, Shandong University of Traditional Chinese Medicine, Jinan, China; ^3^Department of Neurology, Qilu Hospital at Qingdao, Shandong University, Qingdao, China

**Keywords:** ephrin receptor A4, platelet-derived growth factor receptor β, neural progenitor cell, differentiation, hippocampal neurogenesis

## Abstract

Neural progenitor cells (NPCs) have great potentials in cell replacement therapy for neurodegenerative diseases, such as Alzheimer’s disease (AD), by promoting neurogenesis associated with hippocampal memory improvement. Ephrin receptors and angiogenic growth factor receptors have a marked impact on the proliferation and differentiation of NPCs. Although ephrin receptor A4 (EphA4) was shown to directly interact with platelet-derived growth factor receptor β (PDGFRβ), the functional effects of this interaction on neurogenesis in cultured NPCs and adult hippocampus have not yet been studied. Immunoprecipitation demonstrated that EphA4 directly interacted with PDGFRβ in NPCs under ligand stimulation. Ephrin-A1 and PDGF-platelet-derived growth factor BB (BB) significantly increased proliferation and neuronal differentiation of NPCs, which was further augmented by combined treatment of Ephrin-A1 and PDGF-BB. We also found that ligand-dependent proliferation and neuronal differentiation were inhibited by the dominant-negative EphA4 mutant or a PDGFR inhibitor. Most importantly, injection of ephrin-A1 and/or PDGF-BB promoted hippocampal NPC proliferation in the APP/PS1 mouse model of AD, indicating that direct interaction of EphA4 with PDGFRβ plays a functional role on neurogenesis *in vivo*. Finally, studies in NPCs showed that the EphA4/PDGFRβ/FGFR1/FRS2α complex formed by ligand stimulation is involved in neurogenesis *via* ERK signaling. The present findings provided a novel insight into the functional role of direct interaction of EphA4 and PDGFRβ in neurogenesis, implicating its potential use for treating neurodegenerative diseases.

## Introduction

Alzheimer’s disease (AD) is the most common form of dementia in the elderly, characterized by progressive memory impairment, cognitive impairment, and behavioral impairment (Langa, 2015; Yang Y. et al., 2018). The current prevalence of dementia worldwide is estimated at 44.3 million and will triple by 2050. However, there is no cure against this devastating disorder.

Postnatal neurogenesis in mammals occurs in two brain regions: the subgranular zone (SGZ) of the dentate gyrus (DG) in the hippocampus and the subventricular zone (SVZ) of the lateral ventricles. Neural stem cells (NSCs) in the SGZ and SVZ undergo asymmetrical division slowly to generate neural precursors and neuroblasts that migrate for long distances and differentiate into neural cells (Ninkovic and Götz, 2007; Zhao et al., 2008). Altered neurogenesis is considered to be involved in the AD-related cognitive impairment (Bao and Song, 2018). This process is mainly regulated by various factors and signal molecules, including Ephrins and their Eph tyrosine kinase receptors, platelet-derived growth factors (PDGFs) and PDGF receptors (Jiao et al., 2008; Sil et al., 2018). Thus, stimulating of endogenous neurogenesis may promote neuronal regeneration.

Ephrin receptors (Ephs) comprise the largest family of receptor tyrosine kinases and regulate numerous important physiological and developmental processes of the neural stem cells (Cramer and Miko, 2016; Dines and Lamprecht, 2016; Kania and Klein, 2016; Yang J. S. et al., 2018). Based on their specific binding with their ligands, ephrins, Ephs could be divided into two subclasses, including type A and B (Kullander and Klein, 2002). EphA receptors usually bind to glycosylphosphatidylinositol-linked ephrinA ligands, while EphB receptors bind to transmembrane molecules, ephrinBs. However, some EphAs including ephrin receptor A4 (EphA4), can bind to both ephrin-As and ephrin-Bs. The glycosyl phosphatidylinositol linkage in ephrin-As mediate the ligand interactions with the cell membrane, while ephrin-Bs have a short cytoplasmic and transmembrane domain. Several ephrins and their receptors regulate different stages of neurogenesis and are differentially expressed in distinct cell types in the neurogenic niches. EphA4 mainly regulates axon guidance and proliferation of neural stem cells during cortical neurogenesis of the developing and the postnatal brain (North et al., 2009; Khodosevich et al., 2011). Recently, a study has revealed a crucial role for EphA4 in regulating neurogenesis and facilitating efficient neuroblast migration to the olfactory bulbs (OBs; Todd et al., 2017). Forward signaling through EphA4 regulates migration along with organization of neuroblasts and astrocytes in the neurogenic niche (Todd et al., 2017). Thus, EphA4 may protect the brain against neuronal loss, thus playing a neuroprotective role in AD (Willi et al., 2012), Parkinson’s disease (PD; Jing et al., 2012), and other neurodegenerative diseases.

Angiogenic growth factor platelet derived growth factor (PDGF-BB) plays many important roles in regulating proliferation and differentiation of neural progenitor cells (NPCs). In addition to the mitogenic activity, PDGFs have emerged as novel factors for neuronal protection and growth (Sil et al., 2018). Their receptors, PDGFRs, are found to be abundant in neurons and glial cells (Ishii et al., 2017). PDGF-mediated signaling regulates neurogenesis, ligand-gated ion channels, cell survival, synaptogenesis, and development of specific types of neurons in the central nervous system (CNS). PDGF/PDGFR signaling is also related to the pathogenesis of various neurodegenerative diseases through its paradoxical roles in the CNS (Sil et al., 2018).

Our previous study showed that EphA4 and fibroblast growth factor receptors (FGFRs) form a heterodimer, phosphorylate each other when stimulated by ligands, and their interaction promotes proliferation and neurogenesis of mouse embryonic NPCs *via* FGFR substrate 2α (FRS2α) and extracellular regulated protein kinases 1/2 (ERK1/2; Sawada et al., 2015). We also reported that EphA4 and platelet-derived growth factor receptor β (PDGFRβ) formed a heterodimer when they were co-expressed in HEK293T cells and human embryonic stem cell-derived NPCs (Chen et al., 2017). However, the functional role of this interaction on neurogenesis in AD-transgenic mice has not been elucidated. In this study, we examined whether EphA4 and PDGFRβ form a heterocomplex and elucidate their effects on proliferation and differentiation in mouse embryonic NPCs *in vitro* and in adult APP/PS1 transgenic mice brains.

## Materials and Methods

### Reagents

Recombinant human PDGF-BB (cat. no. 220-BB) and recombinant human ephrinA1 fused to human IgG-Fc (ephrinA1-Fc; cat. no. 6417-A1) were used (R&D Systems, Minneapolis, MN, USA). Clustered ephrin-A1-Fc was oligomerized according to the manufacturer’s instructions *via* incubation with recombinant anti-human IgG(Fc) for >1 h at 4°C. The working concentration for clustered ephrin-A1(Fc) and PDGF-BB was 0.5 μg/ml and 20 ng/ml separately as previously reported (Sawada et al., 2015). For injection, 10 ng of PDGF-BB or 0.3 μg of ephrin-A1(Fc) was used in a volume of 2–3 μl as previously reported (Jing et al., 2012) with minor changes. The PDGFR inhibitor STI571 was purchased from Selleck Chemicals.

### Mice and Ethics Statement

APP/PS1 Tg mice and their wild-type (Wt) littermates were purchased from the Model Animal Research Centre of Nanjing University (Stock no. 2010-0001). These animals express the Swedish (K670N/M671L) mutation of human APP together with PS1 deleted in exon nine based on a C57BL/6J background (Han et al., 2019). They were housed in standard cages at an ambient temperature of 22 ± 2°C with 12-h light and 12-h dark cycles, and allowed free access to food and water, until the age of 8 months when they were tested. Aβ deposits in the hippocampus could be detected in 8-month-old Tg mice (Supplementary Figure S2). Genotype was confirmed by PCR of mouse tail tissue, as previously described (Li et al., 2016). All animal experiments were carried out in accordance with the guidelines of the Liaocheng People’s Hospital (Shandong, China) and were approved by the Ethics Committee of Liaocheng People’s Hospital (nos. 201604 and 2017012).

### Cell Culture

HEK293T cells were maintained and passaged in Dulbecco’s modified Eagle’s medium (DMEM) supplemented with 10% fetal bovine serum (FBS). Mouse embryonic NPCs were cultured as previously described (Huang et al., 2017). Briefly, the NPCs obtained from dissected telecephalon on embryonic day 12.5 were passaged (P) as neurospheres in DMEM/F12 (Gibco) supplemented with B27 (Gibco), penicillin/streptomycin (Gibco), FGF2 (Gibco), and epidermal growth factor (EGF; Gibco) for up to three passages (P3). Viral transfection was performed at a multiplicity of infection (MOI) of five on cells that were cultured as neurospheres for 3–5 days. Transfected cells were then dissociated mechanically and seeded onto 96-well plates for proliferation assay or 4-well chamber slides for differentiation assay. More than 95% of the cells were infected with GFP.

For stimulation with ligands, single cells dissociated for P3 neurospheres were adherently cultured and preincubated at 37°C for 5 h before performing a proliferation and differentiation assay in serum-free medium without EGF and FGF2. The inhibitor of PDGFRβ STI571, was added 1 h after starting preincubation to a final concentration of 0.5 μM.

### Reverse Transcription-Quantitative Polymerase Chain Reaction (RT-qPCR)

NPCs were rinsed with RNase-free phosphate buffer saline (PBS) three times after 3 days in culture at 37°C and then incubated in medium devoid of EGF and FGF2 for 5 h, followed by incubation with ligands. The cells were homogenized using TRIzol^®^ reagent (Sigma–Aldrich, St. Louis, MO, USA) to extract RNA according to the manufacturer’s instructions. qPCR analysis was performed with the SYBR Green PCR Master Mix (Applied Biosystems; Thermo Fisher Scientific, Waltham, MA, USA). Three independent experiments were performed in triplicate, with GAPDH as the internal control. Relative expression levels were calculated using the 2^−ΔΔCq^ method (Livak and Schmittgen, 2001). The sequences of the gene-specific primers used are shown in Table 1.

**Table 1:** Primer sequences used in this study.

Primer	Sequence (5′	#x02013;3′)
β III-tubulin-F	CCTTCATCGGGAACAGCACG
β III-tubulin-R	ACTCCTCCTCGTCGTCTTCGTA
GAPDH-F	CAAGGAGTAAGAAACCCTGGACC
GAPDH-R	CGAGTTGGGATAGGGCCTCT

### Plasmid Construction

EphA4, PDGFRβ, FGFR1, and FRS2α eukaryotic expression vectors were constructed as previously described (Yokote et al., 2005; Vanlandewijck et al., 2015). Retrovirus for the dominant-negative mutant of EphA4 containing the deletion of 591–602 amino acids and V635M mutation were harvested using co-transfection of the pMXs-EphA4-IRES-GFP constructs with pCAGVSV-G that encodes vesicular stomatitis virus surface protein under the control of chicken β-actin promoter as previously reported (Yokote et al., 2005).

### DNA Transfection and Immunoprecipitation

Plasmids were transiently transfected into 293T cells using PerFectin (Genlantis). Prior to ligand stimulation, NPCs were starved in serum-free medium containing 0.5% (m/v) bovine serum albumin (Sigma–Aldrich, St. Louis, MO, USA) for 5 h. Immunoprecipitation was performed as previously described (Sawada et al., 2015). Cells were extracted in lysis A buffer with minor modifications as previously described (Sawada et al., 2015), and sonication was performed before BCA protein assay. Following immunoprecipitation with specific antibodies using protein A agarose, the pellets were washed three times. Then, immunoblotting was performed with diluted antibodies for 2 h at room temperature using a standard procedure (Yokote et al., 2005). To confirm reproducibility, experiments were performed at least three times. The following primary antibodies were used: mouse anti-HA (cat. no. 11583816001, Roche), mouse anti-Flag M2 (cat. no. F3165, clone M2; Sigma–Aldrich, St. Louis, MO, USA), mouse anti-myc derived from hybridoma MYC1-9E10.2 (cat. no. CRL-1729, ATCC), mouse anti-phosphotyrosine (clone 4G10, cat. no. 05–321, Millipore, Kankakee, IL, USA), and mouse anti-EphA4 (cat. no. 37–1,600, Thermo Fisher Scientific, Waltham, MA, USA); mouse anti-EphA4 (cat. no. sc-365503), rabbit anti-PDGFRβ (cat. no. sc-432), mouse anti-PDGFRβ (cat. no. sc-374573), rabbit anti-FGFR1 (cat. no. sc-121), mouse anti-GFP (cat. no. sc-9996), rabbit anti-GAPDH (cat. no. sc-25778), and rabbit anti-FRS2a (cat. no. sc-8318) were purchased from Santa Cruz Biotechnology, Santa Cruz, CA, USA; rabbit anti-phosph-p44/42 mitogen activated protein kinase (MAPK; ERK; Thr202/Tyr204; cat. no. 9101), rabbit anti-p44/42 MAPK (ERK; cat. no. 9102), and rabbit anti-FRS2α (Y196; cat. no. 3861) were purchased from Cell Signaling Technology, Danvers, MA, USA. ImageJ software (National Institutes of Health) was used to quantify the band intensities, and the protein interaction or phosphorylation was normalized to the expression of total protein or GAPDH.

### NPC Differentiation and Immunofluorescence

NPC differentiation was performed according to a previously published method with minor changes (Sawada et al., 2015). Briefly, 2 × 10^5^ cells were plated on 4-well chamber slides and incubated at 37°C in 5% CO_2_ with the ligands, PDGF-BB (20 ng/ml) and/or ephrin-A1 (0.5 μg/ml), in growth factor-free medium for 7 days. For immunofluorescence analysis, NPCs were fixed with 4% paraformaldehyde at room temperature for 1 h and blocked in 1% normal goat serum (Sigma–Aldrich, St. Louis, MO, USA) at room temperature for 1 h. The cells were then incubated with mouse anti-Tuj1 antibody (cat. no. T8660; Sigma–Aldrich, St. Louis, MO, USA), mouse anti-Nestin (cat. no. ab22035; Abcam, Cambridge, UK), rabbit anti-Ki67 (cat. no. ab16667, Abcam, Cambridge, UK), rabbit anti-GFAP (cat. no. Z0334, Dako), mouse anti-EphA4 (cat. no. 37–1,600, Thermo Fisher Scientific, Waltham, MA, USA), and rabbit anti-PDGFRβ (cat. no. sc-432) at 4°C overnight, followed by incubation with the secondary antibodies, goat anti-mouse Alexa Flour 488 (cat. no. 115-545-146; Jackson ImmunoResearch Laboratories Inc., West Grove, PA, USA) and goat anti-rabbit cyanine Cy3-conjugated IgG (cat. no. 711-165-152; Jackson ImmunoResearch Laboratories Inc., West Grove, PA, USA) for 2 h at room temperature in the dark. Nuclei were stained with Hoechst 33258 (Santa Cruz Biotechnology, Santa Cruz, CA, USA) at room temperature for 5 min. Cells (four fields per well) were examined under a Nikon fluorescence microscope (Nikon) or a confocal microscope (Olympus).

### Cell Proliferation Assay

Cell proliferation assay was performed based on a previously published method with minor modifications (Huang et al., 2017). Briefly, cells were starved overnight and then 1 × 10^3^ seeded on 96-well plates in culture medium containing growth factors. The ligands, PDGF-BB (20 ng/ml) and clustered ephrin-A1(Fc; 0.5 μg/ml), were added to wells with growth factor-free medium. Cell proliferation was measured after adding 10 μM bromodeoxyuridine (BrdU; Sigma–Aldrich, St. Louis, MO, USA) for 2 h at 37°C according to the manufacturer’s protocol. After culturing for 3 days in refreshed media at 37°C, the cells were fixed with 4% paraformaldehyde at room temperature for 20 min and were denatured in 2N HCl at 37°C for 30 min followed by a wash with 0.1 M Na_2_B_4_O_7_ (pH 8.5). Mouse anti-BrdU (cat. no. MI-11-3, MBL) was used as a primary antibody. Cell proliferation was also measured by a mouse pHH3 (anti-phosphorylated histone H3, cat. no. ab14955, Abcam, Cambridge, UK) antibody following immunocytochemical procedures as shown above. For quantitative image data analysis, all positive-staining cells were counted.

### Stereotactic Surgeries of APP/PS1 Mice

Animals were randomly allocated to five groups: WT control, APP/PS1 mice + PBS, APP/PS1 mice + ephrin-A1 (0.3 μg), APP/PS1 mice + PDGF-BB (10 ng), and APP/PS1 mice + ephrin-A1 plus PDGF-BB. Factors were microinjected into the bilateral hippocampus (AP: 2.06, ML: ±1.75, DV: 1.75) of APP/PS1 mice, and animals were kept alive for another 4 weeks. To assess the differentiation of newborn neurons in the hippocampus, the mice received five daily i.p. injections of BrdU (50 mg/kg mice body weight) each at a 24-h interval and were sacrificed 1 day after the last injection of BrdU.

### Morris Water Maze (MWM) Test

Hippocampal-dependent learning and memory of mice were examined using Morris Water Maze (MWM) following previously standard protocols (Zhang et al., 2014). The MWM consisted of a light-blue swimming pool 160 cm in diameter filled with opaque water maintained at 23 ± 2°C. The SuperMaze system was used to divide the pool into four quadrants [North-West (NW), North-East (NE), South-West (SW), and South-East (SE)] of equal size. During training, mice were paced into the pool facing the walls of each quadrant in the following order: SW, NW, NE, and SE. Each animal underwent four trials per day over five consecutive days, and they were allowed to swim until the escape platform (10 × 10 cm) was found (escape latency) for a maximum of 120 s. The day after completing the 5-day place learning, the mice were placed in the pool but without the escape platform. The time spent in each quadrant was recorded to determine the degree of learning in the animals with respect to where the platform used to be located.

### Immunofluorescence

For the immunofluorescence, the APP/PS1 mice were transcardially perfused with 10 mM PBS 4 weeks after microinjection with multiple factors. One side of the brain was frozen on dry ice and the other side was post-fixed in 4% PFA for 24 h. Subsequently, the fixed brains were cryoprotected in 30% sucrose for 24–48 h, embedded in Tissue-Tek OTC (Sakura Finetek), and sectioned into 20-μm-thick slices with a cryostat (Leica CM1950). One in six sections were incubated in 2 N HCl at 37°C for 30 min, and then 0.1 M Na_2_B_4_O_7_ for another 15 min to neutralize the HCl, followed by immunostaining with BrdU antibody (cat. no. MI-11-3, MBL) and NeuN (cat. no. ABN78, Millipore, Kankakee, IL, USA) or anti-GFAP (cat. no. Z0334, Dako) at 4°C overnight, and incubation with fluorescence-labeled secondary antibodies for 3–4 h at room temperature. Finally, the sections were incubated in nuclear staining Hoechst 33258 (1:10,000) for 2–5 min. All of the images were obtained with a fluorescence microscope (Nikon) or a laser scanning confocal microscope (Olympus). For quantitative image data analysis, all positively stained cells within the SGZ or granule cell layer of the hippocampus DG region were quantified as reported previously (Garza et al., 2008). Briefly, every sixth section from each animal was used to quantify BrdU-positive cells in the SGZ and hillus of the DG bilaterally. The number of BrdU-positive cells per section was calculated and multiplied by six to obtain the total number of cells per DG per mouse.

### Statistical Analysis

Data were analyzed using GraphPad Prism 6 software by two-way analysis of variance followed by Dunnett’s *post hoc* test for multiple comparisons. Difference between two groups was compared using unpaired *t*-test. Data were expressed as the mean ± standard deviation. *p < 0.05, **p < 0.01, ***p < 0.001 was considered to indicate a statistically significant difference.

## Results

### Interaction Between EphA4 and PDGFRβ in Mouse Embryonic NPCs

**Figure 1:**
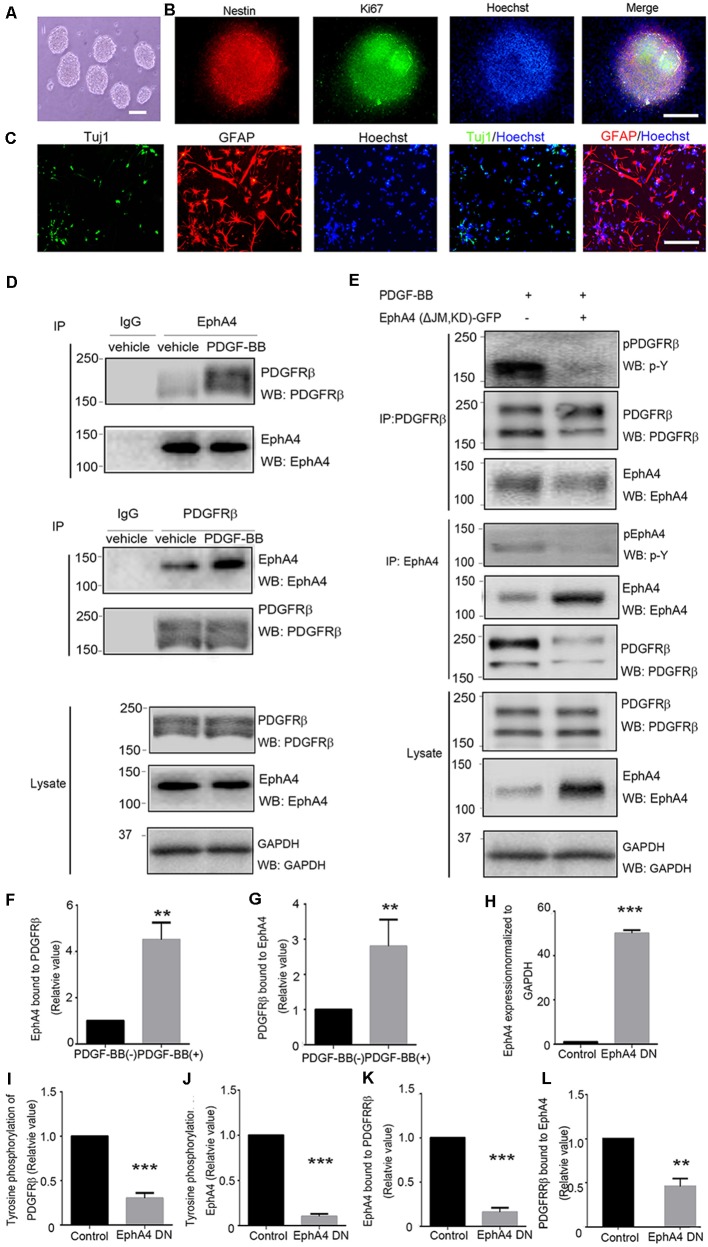
Direct interaction of ephrin receptor A4 (EphA4) and platelet-derived growth factor receptor β (PDGFRβ) in mouse embryonic neural progenitor cells (NPCs). **(A)** Cultured NPCs are amassed as neurospheres. Scale bar = 100 μm. **(B)** Immunofluorescence of the neurospheres shows nestin^+^ and Ki67^+^ labeling. Scale bar = 50 μm. **(C)** NPCs differentiated into neurons (Tuj1^+^) and astrocytes (GFAP^+^). Scale bar = 25 μm. **(D)** The binding of platelet-derived growth factor receptor β (PDGFRβ) with EphA4 and vice versa were examined under platelet-derived growth factor BB (PDGF-BB; 20 ng/ml) stimulation. EphA4 immunoprecipitation was immunoblotted for PDGFRβ or total EphA4, and PDGFRβ immunoprecipitation was immunoblotted for EphA4 or total PDGFRβ. Band intensities were measured using ImageJ software and quantifications of protein interactions were performed **(F,G)**. **(E)** The dominant-negative EphA4 mutant attenuates PDGF-BB-stimulated PDGFRβ/EphA4 interaction and affects PDGFRβ activation. PDGFRβ immunoprecipitation was immunoblotted for p-Y or EphA4. The effect of PDGF-BB on EphA4 activation was determined using immunoprecipitation with EphA4 followed by immunoblotting with a p-Y antibody. Band intensities were measured and quantifications of the overexpression of EphA4 mutant were normalized to GAPDH **(H)**; phosphorylation of PDGFRβ **(I)**, phosphorylation of EphA4 **(J)**, and EphA4/PDGFRβ interaction **(K,L)** were performed. Data are presented as the mean ± standard deviation (*n* = 5 in three independent experiments). **p < 0.01, ***p < 0.001.

We investigated whether EphA4 directly interacted with PDGFRβ in mouse embryonic telencephalon-derived NPCs. NPCs were cultured as neurospheres (Figure 1A) that co-expressed the neural stem cell marker nestin and the proliferation marker Ki67 (Figure 1B). NPCs were able to differentiate into Tuj1^+^ neurons (~4.5%) and GFAP^+^ astrocytes (~50%; Figure 1C). We observed that the EphA4/PDGFRβ interaction in the absence of PDGF-BB stimulation was very weak. However, under PDGF-BB stimulation at 15 min (the peak of ligand stimulation), the EphA4 and PDGFRβ complex was detected in the NPCs through immunoprecipitation and immunoblotting with either anti-PDGFRβ or anti-EphA4 antibodies (Figures 1D,F,G). In the dominant-negative EphA4 mutant-transfected NPCs, the PDGF-BB stimulated EphA4/PDGFRβ interaction and phosphorylation of PDGFRβ or EphA4 were significantly attenuated (all *p* < 0.001; Figures 1E,I–L). Further, we confirmed the overexpression of dominant-negative EphA4 mutant by immunoblotting and compared with the intrinsic EphA4 (*p* < 0.001; Figure 1H), and the expression level was consistent with the previous report (Sawada et al., 2010). These results together with our previous report (Chen et al., 2017) strongly suggested that EphA4 and PDGFRβ could bind to each other directly. The signals mediated by the crosstalk were then detected in mouse embryonic NPCs where both the receptors were expressed.

### Direct Interaction of EphA4 and PDGFRβ in Mouse Embryonic NPCs During Proliferation

**Figure 2:**
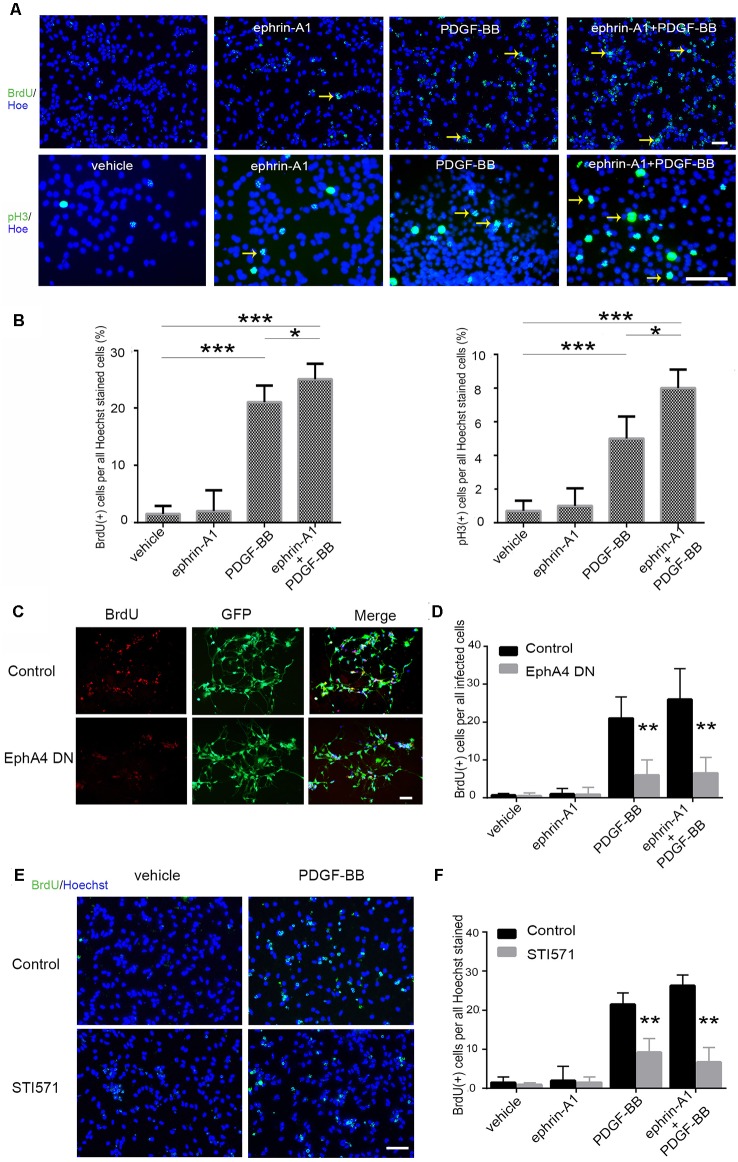
Proliferation of mouse embryonic NPCs under ephrin-A1 and PDGF-BB stimulation. **(A)** BrdU incorporation assay and phospho-Histone H3 (pH3) immunostaining with NPCs plated on a poly-L-lysine and laminin-coated plate. Cells were seeded as a monolayer onto a 96-well plate coated with poly-L-lysine and laminin in normal medium and incubated overnight. The cells were incubated with the indicated reagents [clustered ephrin-A1(Fc), 0.5 μg/ml; PDGF-BB, 20 ng/ml] in a growth factor-free medium for 3 days. For BrdU staining, BrdU (10 μM) was added 2 h before staining. The pictures were taken using a Nikon fluorescence microscope. Arrows indicate the BrdU^+^ (upper panel) or pH3^+^ cells (lower panel). Scale bar = 50 μm. **(B)** Cell proliferation of NPCs were quantitated by calculating the proportion of BrdU^+^ (left panel) or pH3^+^ cells (right panel) among Hoechst^+^ stained cells. **(C,D)** NPCs were transfected with dominant-negative EphA4 mutant prior to stimulation **(C)**. Scale bar = 50 μm. The proportion of BrdU^+^ cells among total infected cells were calculated **(D)**. **(E,F)** NPCs were pretreated with STI571 prior to stimulation **(E)**. Scale bar = 50 μm. The proportion of BrdU^+^ cells among Hoechst^+^ stained cells were calculated **(F)**. Data are presented as the mean ± standard deviation (*n* = 5 in three independent experiments). *p < 0.05, **p < 0.01, ***p < 0.001.

Next, we investigated the functional consequences of the direct interaction between EphA4 and PDGFRβ to understand their role in the proliferation of NPCs. After adding PDGF-BB (20 ng/ml) and/or clustered ephrin-A1(Fc; 0.5 μg/ml) to NPCs, proliferation was analyzed by the BrdU incorporation and the number of phospho-Histone H3 (pH3)^+^ cells (Figure 2A). Statistical analyses showed that PDGF-BB stimulation, but not clustered ephrin-A1(Fc), caused an increase in the BrdU labeling index (the ratio of BrdU^+^ S-phase cells among the Hoechst^+^ cells) and the mitotic index (the ratio of pH3^+^ M-phase cells among the Hoechst^+^ cells) of NPCs when compared with the non-stimulated cells. Furthermore, the ratio of BrdU^+^ cells and pH3^+^ cells further increased under stimulation with clustered ephrin-A1(Fc) and PDGF-BB when compared with non-stimulated cells (both *p* < 0.001) and when compared with just PDGF-BB stimulation (both *p* < 0.05; Figure 2B).

Furthermore, to determine the effect of PDGF-BB and ephrin-A1 on NPC proliferation, retrovirus-mediated EphA4 dominant-negative mutant was expressed in NPCs. BrdU incorporation analysis revealed that expression of the dominant-negative EphA4 mutant markedly inhibited the ligand-stimulated proliferation of NPCs almost to the basal level (Figures 2C,D). Meanwhile, BrdU^+^ cells also decreased significantly when the PDGFR inhibitor STI571 was added to NPCs before ligand stimulation (Figures 2E,F).

### Direct Interaction of EphA4 and PDGFRβ in Mouse Embryonic NPCs During Differentiation

**Figure 3:**
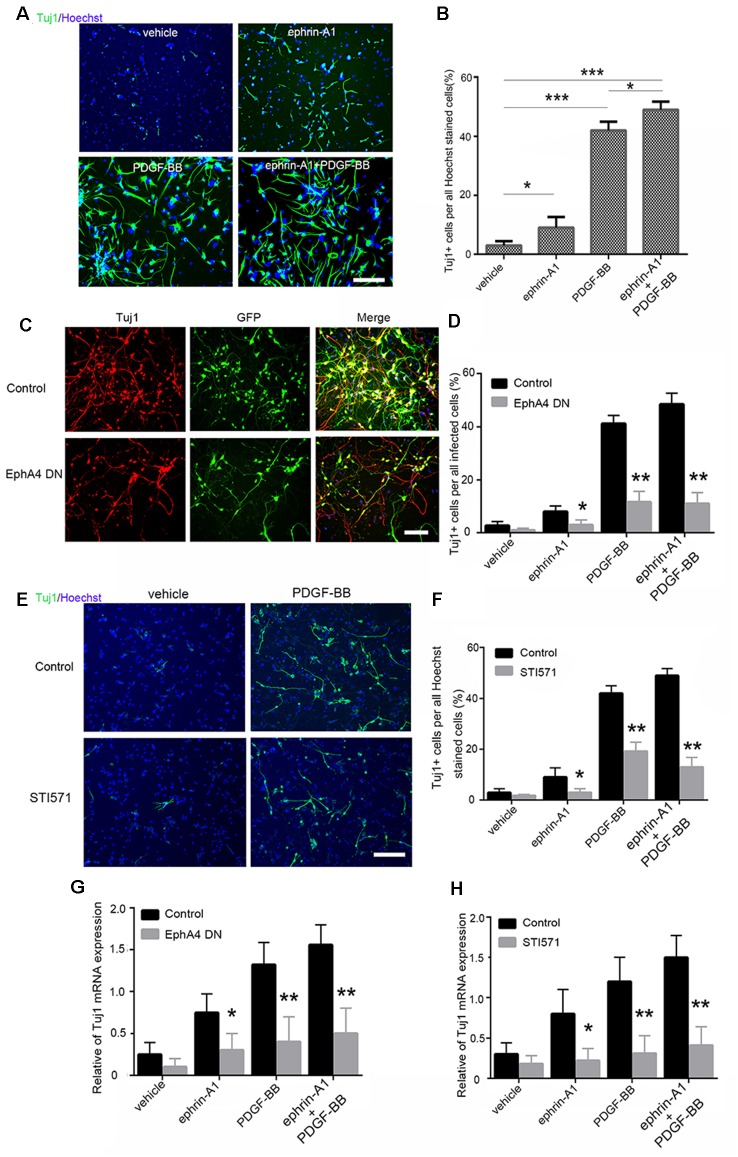
Differentiation of mouse embryonic NPCs under ephrin-A1 and PDGF-BB stimulation. **(A)** NPC differentiation was induced under ephrin-A1 and/or PDGF-BB stimulation. Tuj1^+^ cells in different groups were stained after culturing for 7 days in normal medium or medium containing clustered ephrin-A1(Fc; 0.5 μg/ml) and/or PDGF-BB (20 ng/ml). Scale bar = 100 μm. **(B)** The proportion of Tuj1^+^ cells among Hoechst^+^ stained cells in the different groups were analyzed. **(C,D)** NPCs were transfected with dominant-negative EphA4 mutant prior to stimulation. Representative images of GFP and Tuj1 in NPCs transfected with dominant-negative EphA4 mutant under PDGF-BB stimulation **(C)**. Scale bar = 100 μm. The proportion of Tuj1+ cells among total infected cells were calculated **(D)**. **(E,F)** NPCs were pretreated with STI571 prior to stimulation. Representative images of Tuj1 in NPCs with STI571 under PDGF-BB stimulation **(E)**. Scale bar = 100 μm. The proportion of Tuj1^+^ cells among Hoechst^+^ stained cells in the different groups were calculated **(F)**. **(G,H)** mRNA expression of Tuj1 in NPCs cultured in normal medium or medium containing ephrin-A1 and/or PDGF-BB was also examined. The amount of Tuj1 was obtained by normalizing to GAPDH and presented as the mean ± standard deviation (*n* = 3 in three independent experiments). *p < 0.05, **p < 0.01, ***p < 0.001.

Next, we investigated whether the expression and direct interaction of EphA4 and PDGFRβ serves an important role in the differentiation of NPCs. Immunofluorescence assay demonstrated that the neuroectodermal stem cell marker nestin was expressed in all adherent mouse embryonic NPCs as previously reported (Sawada et al., 2015). Subsequently, PDGF-BB (20 ng/ml) and/or clustered ephrin-A1(Fc; 0.5 μg/ml) were added to these adherent NPCs, and neuronal differentiation was analyzed using Tuj1 immunofluorescence (Figure 3A).

When compared to the non-stimulated cells (3.5%), the proportion of Tuj1^+^ cells increased significantly under stimulation with PDGF-BB (41.5%; *p* < 0.001) and clustered ephrin-A1(Fc; 9%; *p* < 0.05). The ratio of Tuj1^+^ cells exhibited a further increase (49%) under stimulation with clustered ephrin-A1(Fc) plus PDGF-BB when compared with the non-stimulated cells (*p* < 0.001) as well as when compared with just PDGF stimulation (*p* < 0.05), suggesting that enhanced neuronal differentiation of NPCs are induced by simultaneous stimulation with the two ligands (Figure 3B). Furthermore, the ratio of Tuj1^+^ cells markedly decreased in ligand-stimulated NPCs after expression of the dominant-negative EphA4 mutant (Figures 3C,D). Most Tuj1-negative cells in NPCs after expression of the dominant-negative EphA4 mutant were positive for GFAP (Supplementary Figures S1A,B). Meanwhile, the Tuj1^+^ cells also decreased significantly when the PDGFR inhibitor STI571 was added to NPCs before ligand stimulation (Figures 3E,F). Most Tuj1-negative cells in NPCs with STI571 were positive for GFAP (Supplementary Figures S1C,D).

The qPCR analysis indicated that the mRNA level of Tuj1 was markedly upregulated under stimulation with PDGF-BB (*p* < 0.01), clustered ephrin-A1(Fc; *p* < 0.05), and PDGF-BB plus clustered ephrin-A1(Fc; *p* < 0.01) as compared to that of unstimulated control cells. The increased Tuj1 mRNA level in the presence of PDGF-BB and/or clustered ephrin-A1(Fc) was markedly inhibited, with the expression of the dominant-negative EphA4 mutant or with STI571 (Figures 3G,H). Taken together, these results confirmed the role of ephrin-A1 and PDGF-BB during the neuronal differentiation of NPCs.

### Treatment of Ephrin-A1 and/or PDGF-BB Promoted Hippocampal Neurogenesis and Improved Cognitive Impairments in APP/PS1 Transgenic Mice

**Figure 4:**
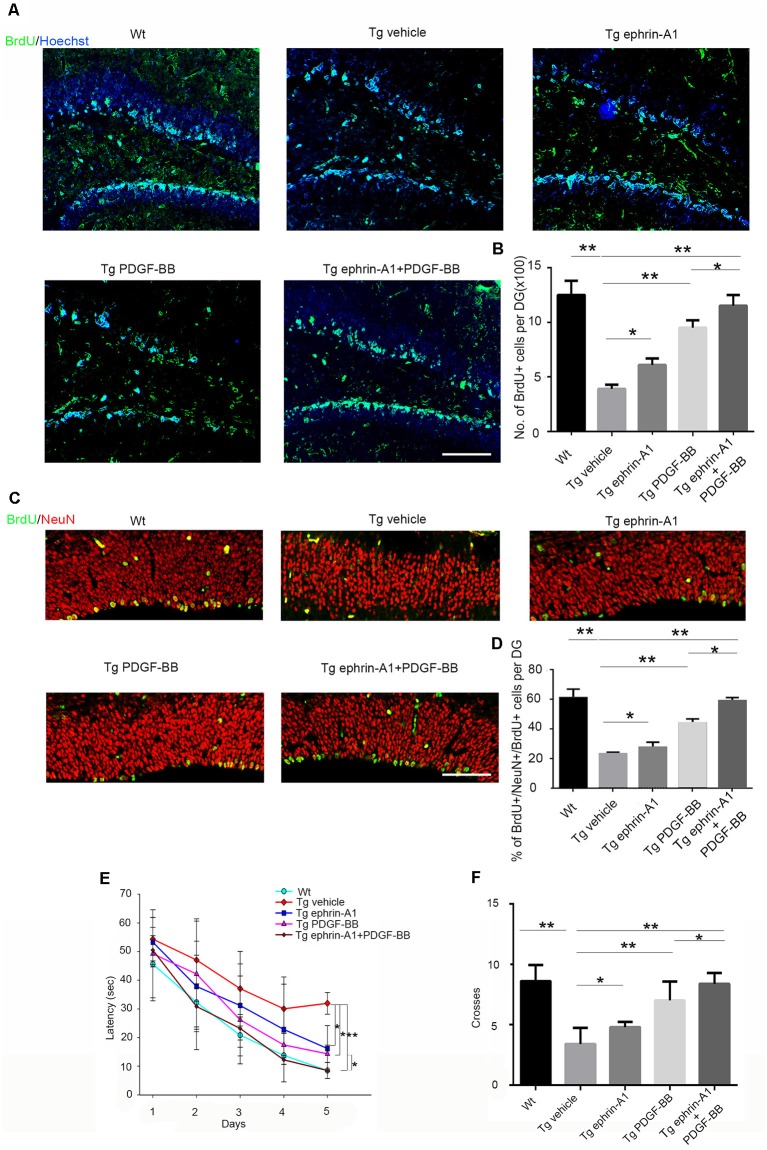
Effect of ephrin-A1 and PDGF-BB on newborn cells in the hippocampus and cognitive deficits in APP/PS1 mice. **(A)** Representative image of BrdU-labeled cells in the dentate gyrus (DG) of hippocampus under the treatment of ephrin-A1 and/or PDGF-BB. Scale bar = 50 μm. **(B)** Quantification of the total number of BrdU^+^ cells in the DG of hippocampus in APP/PS1 mice. All positive-staining cells within the subgranular zone (SGZ) or granule cell layer of hippocampus DG region were counted. Data are presented as the mean ± standard deviation (*n* = 5 per group). *p < 0.05, **p < 0.01. **(C)** Representative images of BrdU/NeuN double-stained cells in the DG of hippocampus under ephrin-A1 and/or PDGF-BB treatment. Scale bar =10 μm. **(D)** Quantification of the proportion of BrdU+ NeuN+ cells among total number of BrdU^+^ cells in the DG of hippocampus in APP/PS1 mice. Data are presented as the mean ± standard deviation (*n* = 5 per group). *p < 0.05, **p < 0.01. **(E)** Morris water maze (MWM) was used 4 weeks after stereotaxic injections to acquit spatial learning. Escape latency means the time for the mouse taken to escape to the platform after it was input in the water. Data are presented as the mean ± standard deviation (*n* = 8–10 per group in three independent experiments). *p < 0.05, **p < 0.01. **(F)**. The number of platform location crosses. Data are presented as the mean ± standard deviation (*n* = 8–10 per group in three independent experiments). *p < 0.05, **p < 0.01.

We further investigated whether ephrin-A1 and PDGF-BB treatment affected neurogenesis using APP/PS1 mouse model of AD. Aβ deposits in the hippocampus could be detected in 8-month-old Tg mice (Supplementary Figure S2). After microinjecting PDGF-BB (10 ng) and/or clustered ephrin-A1(Fc; 0.3 μg) to the hippocampus of APP/PS1 mice, neurogenesis was analyzed by BrdU incorporation (Figure 4A). We found a decrease in BrdU^+^ cells in the DG injected with vehicle of APP/PS1 mice compared with wild-type vehicle-injected mice. The proportion of BrdU^+^ cell in the DG of APP/PS1 animals increased significantly under stimulation with PDGF-BB (*p* < 0.01) and clustered ephrin-A1(Fc; *p* < 0.05) compared with the non-stimulation group. Combined injection with ephrin-A1 and PDGF-BB exhibited a further increase that almost re-established the normal number of BrdU-labeled cells in the DG of APP/PS1 animals compared with the non-stimulation group (*p* < 0.01) and when compared with just PDGF-BB stimulation (*p* < 0.05), suggesting enhanced proliferation of hippocampal NPCs induced by simultaneous stimulation with combined ligands (Figure 4B). To determine the final destiny of newborn cells, we performed double staining of BrdU and NeuN (Figure 4C). The proportion of BrdU+/NeuN+ cells among total BrdU+ cells in the DG of APP/PS1 animals increased significantly under stimulation with PDGF-BB (*p* < 0.01) and clustered ephrin-A1(Fc; *p* < 0.05) compared with the non-stimulation group. Combined injection with ephrin-A1 and PDGF-BB exhibited a further increase in the DG of APP/PS1 animals compared with the non-stimulation group (*p* < 0.01) and when compared with just PDGF-BB stimulation (*p* < 0.05), suggesting enhanced neuronal differentiation of the newborn cells (Figure 4D). Some newborn cells are astrocytes by co-staining of BrdU and GFAP without significant difference between groups (data not shown).

Decreased neurogenesis in the hippocampus DG contributed to memory and cognitive dysfunction. To investigate whether ephrin-A1 and PDGF-BB could improve the learning and memory impairment of APP/PS1 mice, the escape latency of the MWM test was used 4 weeks after stereotaxic injections to evaluate their spatial learning ability (Figure 4E). The MWM results showed that the spatial learning of all the mice was effectively improved during the 5-day training period (*p* < 0.001). Furthermore, we found a significant group effect (*p* < 0.01) on the escape latency. Compared with the wild-type control mice, APP/PS1 mice showed significantly impaired spatial learning ability (*p* < 0.01). The spatial learning ability of APP/PS1 mice improved significantly after separate treatment of PDGF-BB (*p* < 0.05) or clustered ephrin-A1(Fc; *p* < 0.05), and combined ephrin-A1 and PDGF-BB treatment further improved and almost re-established normal memory in mice compared with the non-stimulated group (*p* < 0.01) as well as compared when with PDGF-stimulation (*p* < 0.05; Figure 4E). Finally, these results were confirmed when we analyzed the number of crossings on the location of the platform after its removal to perform the probe trial. Mice in the PDGF-BB or clustered ephrin-A1(Fc) group crossed the removed platform area more often than the vehicle group (*p* < 0.05 for ephrin-A1 group, *p* < 0.01 for PDGF-BB group), and mice in the combined ephrin-A1 and PDGF-BB group crossed the removed platform area further more often than when compared with just the PDGF-BB group (*p* < 0.05; Figure 4F).

### Signaling Mechanisms of Neurogenesis Mediated by EphA4/PDGFRβ Interaction *in vitro*

**Figure 5:**
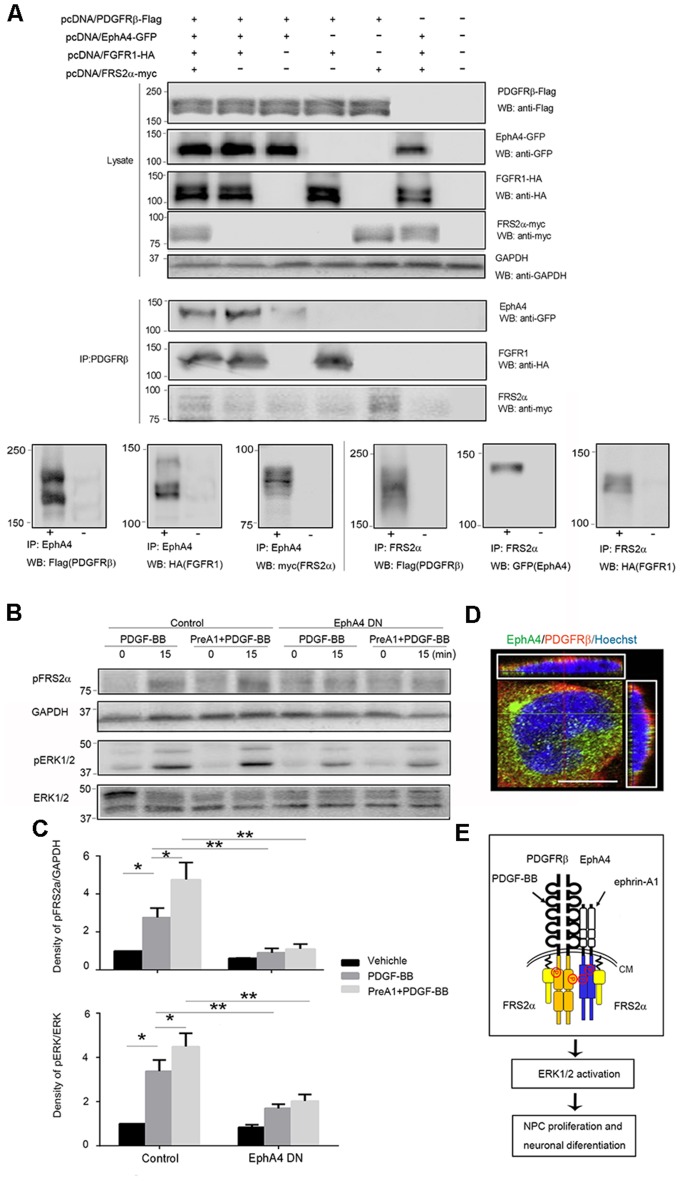
FRS2α and extracellular regulated protein kinases 1/2 (ERK1/2) phosphorylation in response to PDGF-BB and ephrin-A1. **(A)** Complex formation in transfected HEK293T cells. HEK293T cells were co-transfected with pcDNA/PDGFRβ-Flag (1 μg/plate), pcDNA/FGFR1-HA (1 μg/plate), pcDNA/EphA4-GFP (1 μg/plate), and pcDNA/FRS2α-myc (3 μg/plate) in 6-cm plates. Their expression levels using 25 μg of lysates by immunoblotting (WB; upper panels) were detected. Direct interactions were detected by immunoprecipitation (IP) using 150 μg of lysates with anti-PDGFRβ followed by immunoblotting (WB) with antibodies indicated (middle panels) and by immunoprecipitation (IP) with anti-EphA4 (lower left panels) or anti-FRS2α (lower right panels) followed by immunoblotting (WB) with antibodies indicated. For IP shown in lower panels, plasmids co-transfection or vector control was indicated as + and −, respectively. **(B)** Representative Western blot analysis of FRS2α and ERK 1/2 phosphorylation in control and EphA4 dominant-negative transfected mouse embryonic NPCs. GAPDH and total ERK expression were used to show equal sample loading. **(C)** Quantifications of pFRS2α and GAPDH signal intensities or pERK1/2 and total ERK1/2 signal intensities in NPCs stimulated with PDGF-BB with or without 45-min pretreatment of ephrin-A1 (PreA1; bottom). Both ERK1/2 and FRS2α phosphorylation levels were much lower in EphA4 mutant-transfected mouse embryonic NPCs compared to EGFP-transfected controls with the same ligand treatment. Data are presented as the mean ± standard deviation (*n* = 3 per group). *p < 0.05, **p < 0.01. **(D)** EphA4 and PDGFRβ were co-expressed in the cell membrane of NPCs. Scale bar = 25 μm. **(E)** Schematic representation of EphA4 and PDGFRβ complex signaling in NPCs. CM, cell membrane.

To elucidate the mechanisms of the interaction between EphA4 and PDGFRβ, we analyzed the complex formation in mammalian HEK293T cells overexpressing EphA4, PDGFRβ, FGFR1, and FRS2α using immunoprecipitation with anti-PDGFRβ antibody, followed by immunoblotting with anti-EphA4, anti-FGFR1 and anti-FRS2α antibodies. Immunoblotting studies showed expression of PDGFRβ, FGFR1, EphA4, and FRS2α when co-expressed in HEK293T cells (Figure 5A, upper panels). Co-immunoprecipitation and immunoblotting studies showed that PDGFRβ interacted not only with EphA4, but also with FGFR1 and FRS2α (Figure 5A, middle panels). The same was true when EphA4 or FRS2α were immunoprecipitated by specific antibodies (Figure 5A, lower panels). These findings clearly show formation of a bigger complex of PDGFRβ, EphA4, FGFR1, and FRS2α and support the findings that FRS2α associated with PDGFRβ or EphA4 *via* a complex with FGFR1 (Chen et al., 2009; Sawada et al., 2010).

The ERK signaling plays a critical role in proliferation and differentiation of NPCs. Thus, we sought to determine the role of the EphA4/PDGFRβ/FGFR1/FRS2α big complex in neurogenesis. NPCs were transfected with the dominant-negative EphA4 mutant or with GFP control retrovirus, and the activation of FRS2α (pFRS2α), ERK1/2 (pERK1/2) in response to PDGF-BB with or without 45-min pretreatment with ephrin-A1 was examined by Western blotting (Figure 5B). In the EGFP-transfected NPCs, the phosphorylation of FRS2α and ERK1/2 increased in response to PDGF-BB (20 ng/ml) when compared to non-stimulated cells (*p* < 0.05). These cells exhibited an enhanced FRS2α and ERK1/2 activation in response to PDGF-BB after 45-min pretreatment with ephrin-A1 (0.5 μg/ml) compared to PDGF-BB stimulation alone (*p* < 0.05), while in the EphA4 mutant-transfected NPCs, FRS2α and ERK1/2 activation was much less activated in response to ligand stimulation compared to that in EGFP-transfected NPCs, suggesting that reduced EphA4–PDGFRβ interaction diminished FRS2α-ERK signaling in mouse embryonic NPCs (Figure 5C). Fluorescent immunohistochemical analysis also showed that EphA4 and PDGFRβ molecules co-localized on the cell surface of the NPCs (Figure 5D). Collectively, the downstream signaling pathway of the protein complex is schematically shown in Figure 5E.

## Discussion

Neurogenesis declines during aging and neurodegenerative disease process. NPC proliferation is regulated by a variety of extracellular factors and intracellular pathways (Zhao et al., 2008). Stimulating endogenous neurogenesis to restore the cognitive function and memory might be an effective therapeutic strategy for AD (Deng et al., 2010). Here, we identified that combined treatment of eprhin-A1 and PDGF-BB enhanced hippocampal neurogenesis *in vitro* and *in vivo*, which further ameliorated cognitive dysfunctions in APP/PS1 transgenic mice. These responses were the strongest following combined stimulation with eprhin-A1 and PDGF-BB, which could be reversed when either the dominant-negative EphA4 mutant was expressed or a PDGFR-inhibitor was applied. EphA4 and PDGFRβ form a complex that is involved in transducing signals through the EphA4–PDGFRβ–FGFR1–FRS2α complex and ERK1/2 pathway.

We have found a direct interaction between EphA4 and PDGFRβ in NPCs. This result is consistent with a previous study showing a direct interaction between them in HEK293T cells after overexpression of EphA4 and PDGFRβ (Chen et al., 2017). In this report, EphA4 dominant-negative mutant showed a strong inhibitory effect on PDGF-BB treatment of BrdU^+^ cells and Tuj1^+^ cells almost to the basal level; this is probably because EphA4 dominant-negative mutant inhibited both the PDGF-mediated and the ephrin-mediated signaling. Together with previous findings showing that EphA4 dominant-negative mutant inhibited EphA4 and PDGFRβ activation at both basal and ligand-stimulated conditions (Sawada et al., 2010; Chen et al., 2017), these results provided evidence of direct molecular interactions between EphA4 and PDGFRβ.

We previously reported that a direct interaction and trans-activation of EphA4 and FGFRs activates a downstream target, FRS2α, which further phosphorylates ERK1/2 in mouse embryonic NPCs and in EphA4-deleted mutant mice (Chen et al., 2015; Sawada et al., 2015). Consistent with our previous report showing mitogenic activity, heterocomplex signals were shown to mediate augmentation of NPC proliferation and differentiation ability when stimulated by both ephrin-A1 and PDGF-BB. Combined stimulation with ephrin and PDGF induced MAP kinase activation caused by activation of FGFR adaptor protein, FRS2α. These signals resulted in promotion of NPC proliferation and differentiation activity.

PDGFRs and PDGFs are expressed in several cell types including brain cells such as neurons, astrocytes, oligodendrocytes, microglia, and neural progenitors. PDGF-mediated signaling has been proven to play important roles in neurogenesis, modulation of ligand-gated ion channels, cell survival, development of specific types of neurons, and synaptogenesis in the CNS. Activation of PDGF/PDGFR signaling could elicit positive roles in CNS development and vascularization, relying on the activation stimuli and the cell type, and could be developed as potential targets for treatment of CNS development deficits and abnormal vascularization (Sil et al., 2018). PDGF-BB induces activation of FGFR-1, which is required for a full mitogenic response to PDGF-BB. PDGF-BB and FGF2 reciprocally increases their endothelial cell and mural cell responses (Nissen et al., 2007; Vanlandewijck et al., 2015).

The function of EphA4 in neurogenesis and neurodegeneration remains controversial. The activity of EphA4 was reported to promote neurodegeneration in several models. EphA4 activation mediates synaptic damage in AD (Vargas et al., 2014; Zhao et al., 2018) and its inhibition rescues neurodegeneration in amyotrophic lateral sclerosis (ALS; Faruqi, 2012; Van Hoecke et al., 2012). EphA4 regulates proliferation of neural stem cells during cortical neurogenesis of the developing and the postnatal brain (North et al., 2009; Khodosevich et al., 2011). The current study focused on the promotion of neurogenesis mediated by ephrin-A1 and PDGF-BB stimulation, while the studies by Vargas et al. (2014) focused on the genetic modulation of EphA4 signaling. The results of the current study are consistent with the report that EphA receptor function as positive regulators of the mitogen-activated protein kinase signaling pathway to induce neurogenesis of neural precursor cells from the developing CNS (Aoki et al., 2004; Sawada et al., 2015).

FRS adaptor proteins, which are dedicated to neurotrophin and FGF signaling, have been recently reported to play important roles in developmental neurogenesis in the hippocampus, as well as in dendritogenesis in developmentally generated dentate granule cells (Nandi et al., 2018). The novel finding in this study was that the mitogen-activated protein kinase signaling pathway associated with these signals was mediated by the EphA/PDGFR/FRS2α complex.

The results of the present study demonstrated that a combination of ephrin-A1 and PDGF-BB promoted the proliferation of mouse NPCs and the expression of neuronal marker Tuj1. This combined treatment restored the cognitive function and memory through stimulation of hippocampal endogenous neurogenesis in APP/PS1 model mice. While this is one possible model, other possibilities exist, which have not been ruled out: non-autonomous effect of EphA4, separate complexes at the plasma membrane interact at the level of endocytic vesicles rather than plasma membrane, and so on. Further, we plan to transplant NPCs pretreated with ephrin-A1 and PDGF-BB into animal models as a potential treatment for neurodegenerative diseases such as AD.

Taken together, these results of our study demonstrated that interaction of EphA4 and PDGFRβ promoted neurogenesis, which might be beneficial for treatment of memory loss in AD.

## Data Availability Statement

The raw data supporting the conclusions of this article will be made available by the authors, without undue reservation, to any qualified researcher.

## Ethics Statement

The animal study was reviewed and approved by Ethics Committee of Liaocheng People’s Hospital.

## Author Contributions

QC and FH conceived, designed the experiments and wrote the article. QC, HS, CL, JX, WW, and CW performed the experiments and analyzed the data. All authors approve of this submission in its current version.

## Conflict of Interest

The authors declare that the research was conducted in the absence of any commercial or financial relationships that could be construed as a potential conflict of interest.

## Abbreviations

EphA4: ephrin receptor A4
PDGFRβ: platelet-derived growth factor receptor β
PDGF-BB: platelet-derived growth factor BB
VEGFR2: vascular endothelial growth factor receptor 2
NPC: neural progenitor cell
AD: Alzheimer’s disease
PD: Parkinson’s disease
ALS: amyotrophic lateral sclerosis
FGFR: fibroblast growth factor receptor
FRS2α: FGFR substrate 2α
Tuj1: class III β-tubulin
ERK1/2: extracellular regulated protein kinases 1/2
MOI: multiplicity of infection
BrdU: bromodeoxyuridine
pH3: phospho-Histone H3
PBS: phosphate buffer saline
DG: dentate gyrus
SGZ: subgranular zone
SVZ: subventricular zone.
