# Supplementary material for: The Interaction of EphA4 With PDGFRβ Regulates Proliferation and Neuronal Differentiation of Neural Progenitor Cells in vitro and Promotes Neurogenesis in vivo
Source: Front Aging Neurosci. 2020 Feb 11;12:7. doi: 10.3389/fnagi.2020.00007 (PMC7026009; doi:10.3389/fnagi.2020.00007)
Supplement: Supplementary file 1 [file Data_Sheet_1.pdf]

Supplementary Material

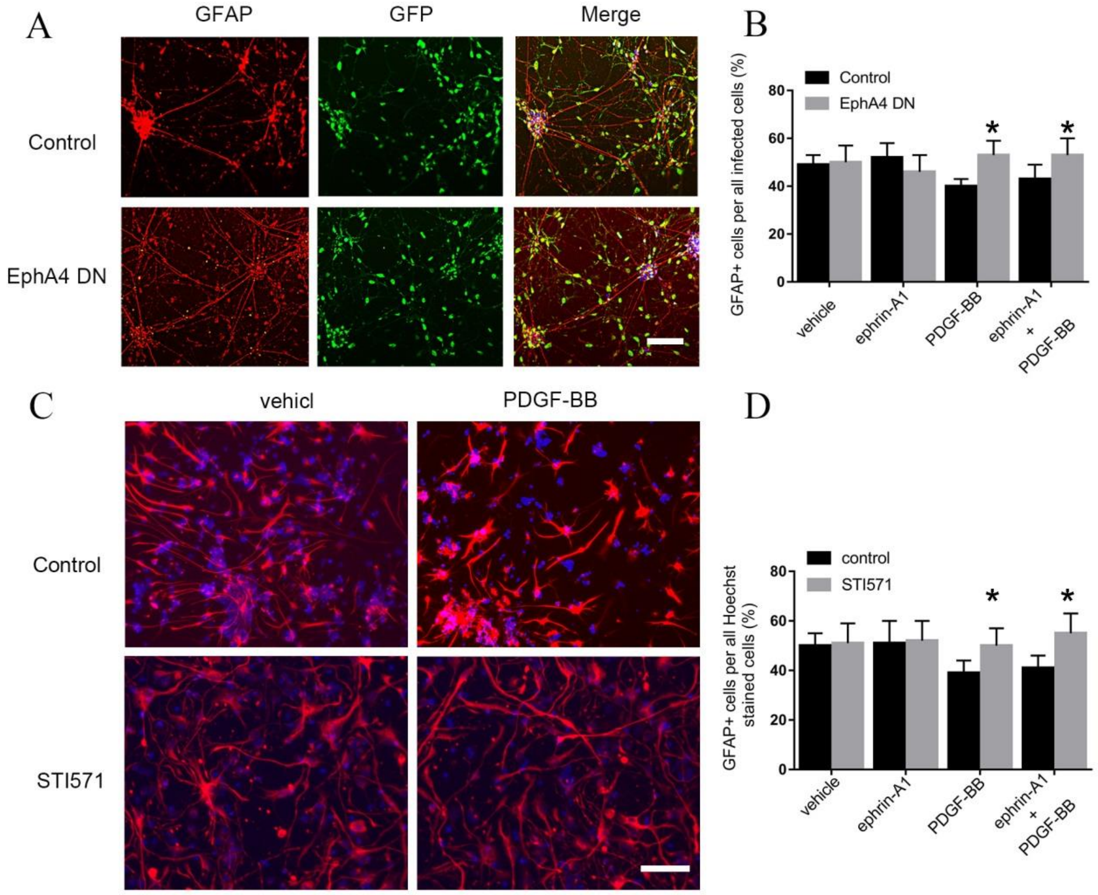

Supplementary Figure 1. Differentiation of mouse embryonic NPCs under ephrin-A1 and PDGF-BB stimulation. (A, B) NPCs were transfected with dominant-negative EphA4 mutant prior to stimulation. Representative images of GFP and GFAP in NPCs transfected with dominant-negative EphA4 mutant under PDGF-BB stimulation (A). Scale bar = 100 μm. The proportion of GFAP+ cells among total infected cells were calculated (B). (C, D) NPCs were pretreated with STI571 prior to stimulation. Representative images of GFAP in NPCs with STI571 under PDGF-BB stimulation (C). Scale bar = 100 μm. The proportion of $GFAP^{+}$ cells among $Hoechst^{+}$ stained cells in the different groups were calculated (D). Data are presented as the mean ± standard deviation (n = 5 in three independent experiments). $^{*}p < 0.05$

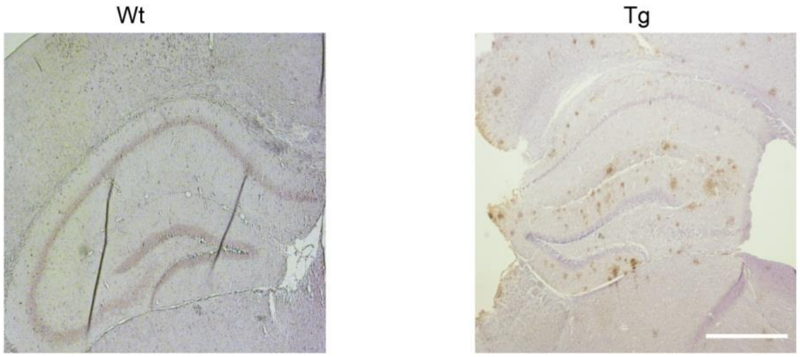

Supplementary Figure 2. Aβ deposits in the hippocampus by Aβ1-42 immunohistochemistry. Aβ deposits could be detected in the hippocampus of 8-month-old Tg mice but not in the hippocampus of Wt littermates. Scale bar = 50 μm.
